# Comorbidities in Recent-Onset Adult Type 1 Diabetes: A Comparison of German Cohorts

**DOI:** 10.3389/fendo.2022.760778

**Published:** 2022-06-03

**Authors:** Oana P. Zaharia, Stefanie Lanzinger, Joachim Rosenbauer, Wolfram Karges, Karsten Müssig, Sebastian M. Meyhöfer, Volker Burkart, Michael Hummel, Dirk Raddatz, Michael Roden, Julia Szendroedi, Reinhard W. Holl

**Affiliations:** ^1^ Institute for Clinical Diabetology, German Diabetes Center, Leibniz Center for Diabetes Research at Heinrich Heine University, Düsseldorf, Germany; ^2^ German Center for Diabetes Research (DZD), München-Neuherberg, Germany; ^3^ Department of Endocrinology and Diabetology, Medical Faculty, Heinrich Heine University, Düsseldorf, Germany; ^4^ Institute of Epidemiology and Medical Biometry, Zentralinstitut für Biomedizinische Technik (ZIBMT), University of Ulm, Ulm, Germany; ^5^ Institute for Biometrics and Epidemiology, German Diabetes Center, Leibniz Center for Diabetes Research at Heinrich Heine University, Düsseldorf, Germany; ^6^ Division of Endocrinology and Diabetes, Rheinisch-Westfälische Technische Hochschule (RWTH) Aachen University, Aachen, Germany; ^7^ Department of Internal Medicine/Gastroenterology, Franziskus-Hospital Harderberg, Georgsmarienhütte, Germany; ^8^ Institute for Endocrinology and Diabetes, University of Lübeck, Rosenheim, Lübeck, Germany; ^9^ Diabetes Center Rosenheim, Rosenheim, Germany; ^10^ Division of Gastroenterology and Gastrointestinal Oncology and Endocrinology, University Medical Center Göttingen, Göttingen, Germany; ^11^ Department of Internal Medicine I and Clinical Chemistry, University Hospital Heidelberg, Heidelberg, Germany

**Keywords:** dyslipidaemia, hypertension, type 1 diabetes, complications, nephropathy, retinopathy

## Abstract

**Aims:**

Restrictive exclusion criteria from different study populations may limit the generalizability of the observations. By comparing two differently designed German cohorts, we assessed the prevalence of cardiovascular risk factors and diabetes-related complications in recent-onset adult type 1 diabetes.

**Methods:**

This study evaluated 1511 persons with type 1 diabetes of the prospective diabetes follow-up registry (DPV) and 268 volunteers of the prospective observational German Diabetes Study (GDS) with a known diabetes duration <1 year. Participants had similar age (36 years), sex distribution (41% female) and BMI (26 kg/m^2^) in both cohorts.

**Results:**

The average HbA1c was 6.4 ± 0.8% in the GDS and 7.0 ± 1.1% in the DPV. Prevalence of hypertension (24%) was similar, while more DPV participants had dyslipidemia and lipid-lowering medication than GDS participants (77% vs. 41% and 7% vs. 2%, respectively; p<0.05). Prevalence of retinopathy and nephropathy was higher in DPV compared to GDS participants (10% vs. 3% and 18% vs. 7%, respectively; p<0.001).

**Conclusions:**

Diabetic nephropathy and retinopathy are the most frequent complications in type 1 diabetes, affecting up to every 10th patient within the first year after diagnosis, underlining the need for more stringent risk factor management already at the time of diagnosis of type 1 diabetes.

## Introduction

The prevalence of comorbidities such as hypertension and dyslipidemia is often underestimated in patients with newly diagnosed type 1 diabetes (1, 2). However, a growing body of evidence emphasizes the frequent existence of cardiovascular risk factors also in newly manifested type 1 diabetes and underlines the need for effective cardioprotective measures from disease onset (2).

Seminal studies demonstrated that intensive glucose lowering therapy could reduce the incidence of microvascular complications (3). Consequently, preventive and therapeutic efforts have focused on optimizing glycemic control (4). Nevertheless, the discordance observed between glucose lowering treatment regimens and the prevalence of different complications suggests distinct risk factors associated with each of the diabetes-related complications (5). Hyperglycemia, hypertension, and dyslipidemia are well defined modifiable risk factors that promote the appearance of micro- and macrovascular complications in diabetes (6). Prompt and intensive treatment are critical for successful prevention or delay of micro- and macrovascular complications (7). However, treatment targets are frequently not met (8) and there is a paucity of data regarding sex specific differences in patterns of diabetes-related complications (9) in patients with type 1 diabetes.

Evidence from population-based studies addressing risk factors and predictors of severe complications can contribute to guidelines for clinical practice and large-scale management of diabetes (10, 11). Moreover, patient selection is crucial to the generalizability of the results of clinical studies on the distribution of risk factors and complications within the diseased population. Early detection and treatment of comorbid conditions is important as it has been indicated that health-related quality of life is preserved in persons with type 1 diabetes unless confronted with multiple comorbidities. Previous findings show the importance of tracking the presence of multiple comorbid conditions, by reviewing medical records, as well as screening for complications in routine diabetes care (12). Our study, therefore, assesses the risk profiles of voluntary study participants of a comprehensive longitudinal clinical study on one side and, on the other side, patients from a patient registry. This comparative evaluation of differently designed German cohorts gives the opportunity to focus on modifiable risk factors that can impact the development of complications in patients with type 1 diabetes in finer detail.

The aim was to compare the prevalence of comorbidities such as hypertension and dyslipidemia and the presence of diabetes-related complications in patients with recently diagnosed type 1 diabetes from the prospective cohort of the German Diabetes Study (GDS) and from the national diabetes prospective follow-up registry (DPV) and assess sex-specific differences in diabetes-related complications.

## Subjects, Materials and Methods

### Data Sources

We analyzed data from two cohorts, the GDS and the DPV. GDS is a prospective longitudinal observational multicenter cohort study investigating the phenotypes of diabetes and diabetes-related comorbidities and complications during the course of the disease (13). In addition to demographic and clinical data, baseline and follow-up assessments include deep metabolic phenotyping in order to identify prognostic factors and underlying mechanisms contributing to diabetes progression and the development of its comorbidities and complications. The GDS (Clinicaltrials.gov NCT01055093) was approved by the ethics boards of the Medical Faculty of the Heinrich Heine University Düsseldorf (reference number 4508) and is being performed according to the Declaration of Helsinki (13).

The DPV registry is an electronic health record-based documentation for both children and adults with any diabetes type. Relevant data pertaining to diabetes are documented by the physician/diabetes nurse specialist and are available for research purposes. Anonymized demographic and clinical data from patients are transmitted for central validation and analyses to Ulm University. The ethics committee of Ulm University as well as local review boards of the participating centers approved data collection and analysis for benchmarking and diabetes research. Data from 265 specialized centers in Germany, including 39 university hospitals or out-patient clinics and 226 private practices were studied (14). A rough estimate indicates that almost a quarter of German adults with diabetes are registered in the DPV.

### Participants

Patients aged 18-69 years with newly diagnosed type 1 diabetes (disease duration <12 months) (13) from 2009 to 2017 were included. Diagnosis of diabetes was confirmed according to the criteria of the current guidelines: fasting plasma glucose ≥ 126 mg/dl or 2-hour plasma glucose during oral glucose tolerance test ≥ 200 mg/dl or HbA1c ≥ 6.5% (15, 16). Specific criteria for type 1 diabetes such as severe hyperglycemia and/or diabetic ketoacidosis as well as low or undetectable levels of plasma C-peptide were considered (15, 16). Exclusion criteria comprised specific types of diabetes due to other causes, including hereditary forms of diabetes (e.g. maturity onset diabetes of the young, MODY), pregnancy or poor glycemic control (HbA1c >9%) (13). For the GDS cohort, additional exclusion criteria were severe renal diseases (stage 3 and above; estimated glomerular filtration rate (eGFR) <60 ml*min^-1^*1.73m^-^²) and evidence for acute inflammatory syndromes (high-sensitivity C-reactive protein (hsCRP) >1 mg/dl) (17).

### Risk Factors and Comorbidities

Patients’ medical history, current medication as well as laboratory and clinical parameters were recorded. Blood pressure was measured in resting conditions. In patients of the GDS, serum levels of blood glucose, HbA1c, serum lipids (total cholesterol, LDL- and HDL-fractions and triglycerides) and creatinine were measured in fasted conditions as previously described (13). In the DPV cohort, analyses of blood parameters were performed in each center following standardized operating procedures (18).

Dyslipidemia was initially defined by serum levels of triglycerides were ≥150 mg/dl and/or total cholesterol ≥200 mg/dl and/or HDL-cholesterol ≤40 mg/dl and/or LDL-cholesterol ≥160 mg/dl. Thereafter, as newest guidelines (European Society of Cardiology, ESC) advocate for a stratified risk assessment with individualized targets for LDL cholesterol (19), the proportion of persons meeting these targets was also assessed. Hypertension was defined as systolic blood pressure ≥140 mmHg and/or diastolic blood pressure ≥90 mmHg.

Patient self-reported history of diabetes-associated complications such as myocardial infarction, stroke, diabetic foot syndrome and peripheral artery disease was recorded. In the DPV diagnosis of myocardial infarction, stroke, diabetic foot syndrome and peripheral artery disease is based on physician diagnosis (ICD based). Eye examinations were performed and evaluated by trained ophthalmologists differentiating between non-proliferative and proliferative retinopathy in accordance with ADA guidelines (20). Albuminuria was assessed as marker of diabetic nephropathy. Urine samples were analyzed and microalbuminuria was defined by urinary albumin levels between 30-300 mg/l and macroalbuminuria by albumin levels above 300 mg/l (21).

### Statistical Analyses

Descriptive statistics are presented for both cohorts. Continuous variables are reported as mean ± SD and dichotomous variables as percentages. Kruskal-Wallis and chi-squared tests were performed to identify unadjusted differences between the GDS and DPV patient groups. Benjamini-Hochberg procedure was used to control the false discovery rate and to adjust for multiple testing. Event rates of acute complications per 100 patient years (PY) were calculated using Poisson regression models. Multivariable logistic regression models were used in order to evaluate differences in the presence of diabetes-related complications between the two cohorts. All models were adjusted for sex, age groups (<30, 30-<50, ≥50 years), BMI (categorized as <25, 25-<30, ≥30 kg/m^2^) and migration background (at least one parent not born in Germany). A subsequent analysis additionally adjusting for hypertension and dyslipidemia was performed to study whether these parameters are associated with the presence of diabetes-related complications. Additionally, a pooled analysis of the GDS and DPV cohort was performed to identify sex-related differences in diabetes complications as well as age-stratified analyses for age groups (<30, 30-<50, ≥50 years). P-values for two-sided tests below 0.05 were considered to indicate statistical significance and analyses were conducted with SAS version 9.4 (SAS Institute Inc, Cary, NC, USA).

## Results

**Figure 1:**
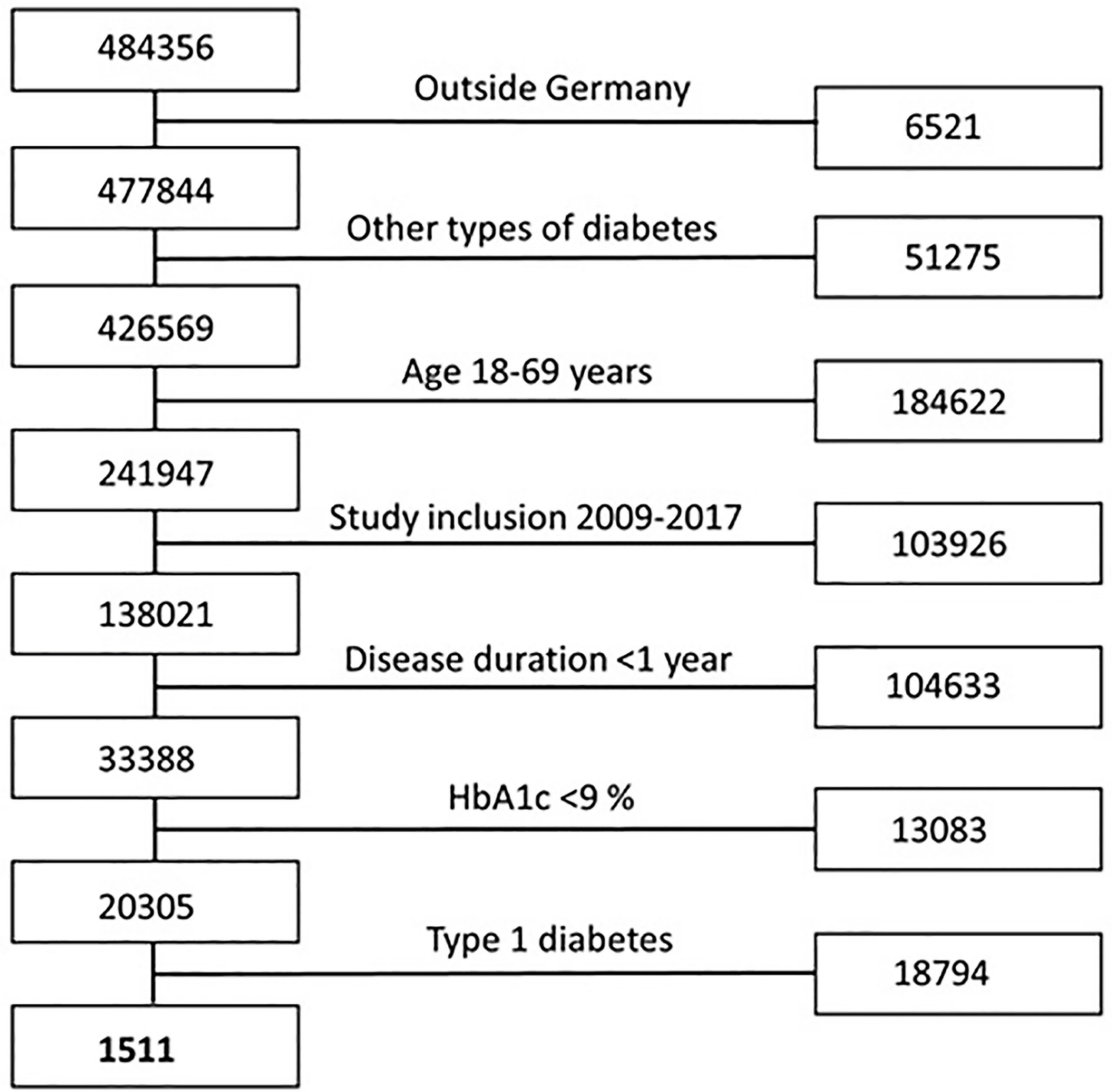
Flow chart participants’ selection criteria for the DPV registry. Flow chart showing the included and excluded participants of the DPV registry based on selection criteria matching the inclusion criteria of the GDS.

In total, 1779 patients (268 participants of the GDS and 1511 patients of the DPV registry) (**Figure 1**) were included and analyzed with regard to metabolic phenotypes, routine laboratory parameters, clinical data including disease duration, anthropometric variables, medication, the presence of diabetes-related comorbidities and complications and smoking status.

### Anthropometry and Clinical Parameters

The baseline information on demographic variables, clinical parameters and behavioral risk factors is shown in **Table 1**.

**Table 1:** Anthropometric and clinical parameters of patients with newly diagnosed type 1 diabetes from the GDS and DPV cohorts.

	GDS		DPV		p-value
Variable	n		n		
Sex [% male]	268	59.3	1511	58.7	0.848
Age [years]	268	36.5 ± 11.6	1511	35.6 ± 14.6	0.050
Known diabetes duration [years]	268	0.52 ± 0.24	1511	0.38 ± 0.31	<0.001
Migration-background [%]	267	10.9	1511	6.3	0.020
Current smokers [%]	147	39.5	1109	28.2	0.007
Cigarettes/day [n]	44	12.9 ± 16.5	1109	3.7 ± 7.3	<0.001
BMI [kg/m^2^]	268	25.0 ± 4.3	1443	24.9 ± 5.2	0.436
Systolic blood pressure [mmHg]	264	128.8 ± 16.3	1424	125.3 ± 14.8	0.003
Diastolic blood pressure [mmHg]	264	77.4 ± 9.7	1423	76.2 ± 10.4	0.239
Positivity for diabetes-related autoantibodies [%]	267	92.5	598	88.8	0.140
Fasted blood glucose [mg/dl]	264	129.1 ± 36.7	860	152.0 ± 88.8	<0.012
HbA1c [%]	267	6.4 ± 0.8	1511	7.0 ± 1.1	<0.001
HbA1c [mmol/mol]	267	46 ± 9	1511	53 ± 12	<0.001
Total cholesterol [mg/dl]	268	184.8 ± 38.4	926	190.0 ± 49.9	0.315
LDL-cholesterol [mg/dl]	267	110.4 ± 33.4	861	112.1 ± 36.5	0.606
HDL-cholesterol [mg/dl]	267	60.7 ± 17.1	874	55.4 ± 19.1	<0.001
Triglycerides [mg/dl]	268	89.7 ± 57.2	837	138.3 ± 114.1	<0.001
ALT [U/l]	268	21.9 ± 7.3	258	27.7 ± 31.7	0.108
AST [U/l]	268	24.0 ± 13.4	390	29.6 ± 27.2	0.018
GGT [U/l]	268	22.0 ± 21.2	376	53.3 ± 157.0	<0.001
Creatinin [mg/dl]	268	0.9 ± 0.2	1116	0.85 ± 0.53	<0.001
TSH [µlU/ml]	266	2.5 ± 2.6	984	0.9 ± 2.2	<0.001

n columns refer to the number of examined patients for each parameter. Data are shown as absolute numbers, percentages, mean ± standard deviation, as applicable. ALT, alanine aminotransferase; AST, aspartate aminotransferase; BMI, body mass index; DPV, Prospective Diabetes Registry; GDS, German Diabetes Study; GGT, gamma-glutamyl transferase; HbA1c, glycated hemoglobin A1c; HDL, high-density lipoprotein; LDL, low-density lipoprotein; TSH, thyroid stimulating hormone.

Patients with type 1 diabetes did not differ between cohorts with regard to age, sex distribution or BMI. In DPV, 61% of participants had a BMI <25 kg/m^2^, 26% were overweight (BMI 25-30 kg/m^2^) and 13% were obese (BMI >30 kg/m^2^). In GDS, 31% participants were overweight and 10% were obese.

While the GDS routinely screens for diabetes-associated autoantibodies, only 39% of the persons with type 1 diabetes in the DPV were investigated regarding diabetes-related autoimmunity. Diabetes-associated autoantibodies were detected in 92.5% of the participants with type 1 diabetes of the GDS and in 88.8% of the investigated patients of the DPV cohort.

Participants of the GDS were more frequently smokers, and consumed a higher number of cigarettes per day compared to patients of the DPV (**Table 1** all p<0.05). Of note, smoking habits were not systematically registered in both study cohorts (GDS 55%, DPV 73%).

### Hyperglycemia and Glucose-Lowering Medication

**Figure 2:**
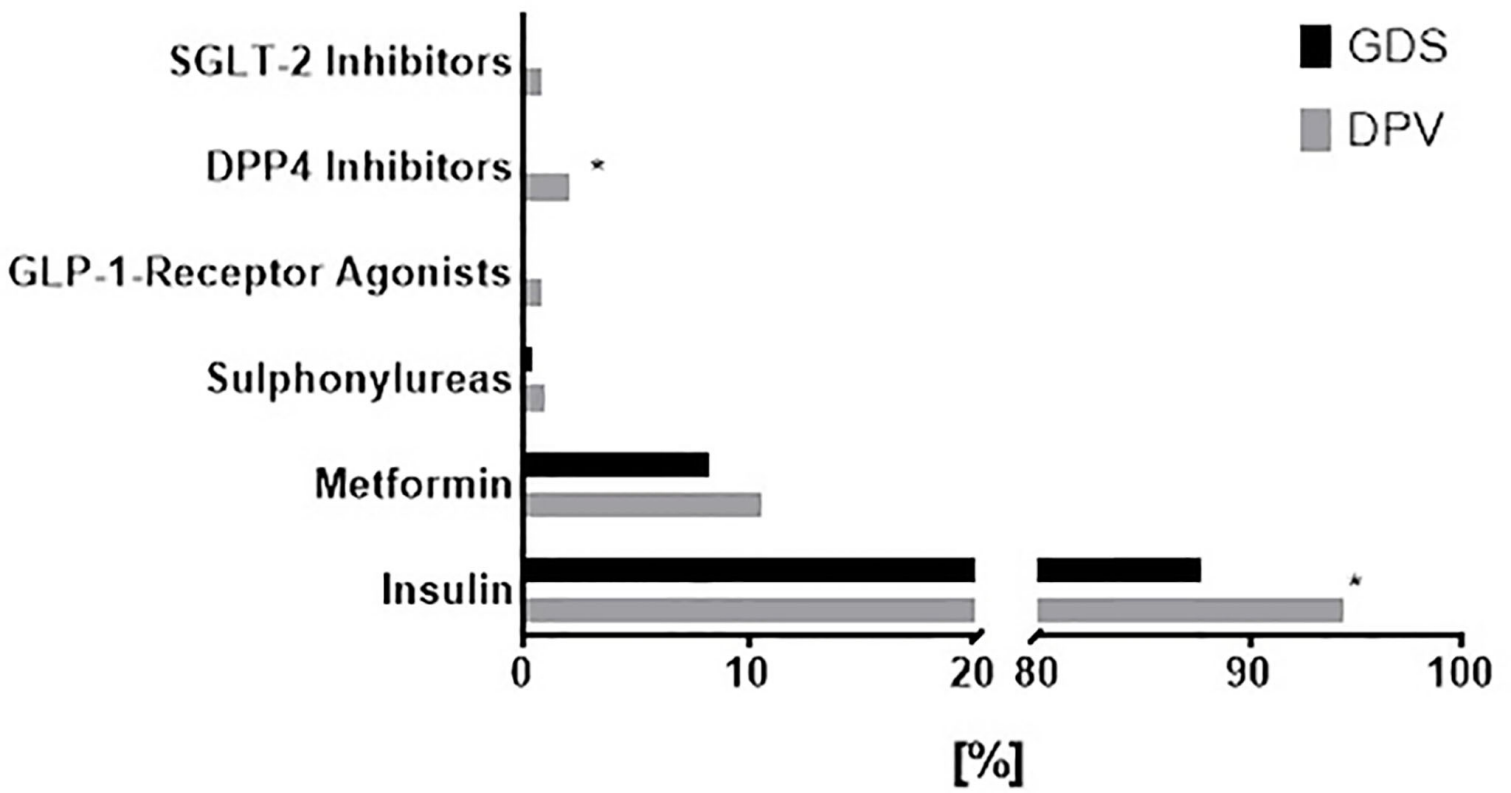
Glucose-lowering medication in patients with newly diagnosed type 1 diabetes from the GDS and DPV cohorts. Choice of glucose-lowering medication in patients with newly diagnosed type 1 diabetes from the GDS (black) and DPV (grey) cohort. Data was collected on the frequency of use of sodium-glucose co-transporter-2 (SGLT-2) inhibitors, dipeptidyl peptidase-4 (DPP4) inhibitors, glucagon-like peptide-1 (GLP-1) receptor agonists, sulphonylureas, metformin and insulin. Data are expressed as percent from the total number of participants in each cohort. P values refer to comparison of unadjusted data. *p < 0.05.

Only patients with HbA1c <9% (75 mmol/mol) were included in this analysis in order to match for the inclusion criteria of the GDS (13). Of these individuals, 54% from DPV and 88% of GDS had good glycemic control (HbA1c <7%/53 mmol/mol). Current glucose-lowering medication is shown in **Figure 2**. As expected, newly diagnosed patients with type 1 diabetes were preferentially treated with insulin. In 14% of patients additional oral glucose-lowering medication was recorded. Metformin was the most frequently used oral therapy.

In the DPV registry event rates of acute complications were 13.8 events/100 patient years (PY) (95% confidence interval: 12.7-14.9) of severe hypoglycemia, 4.6 events/100 PY (4.0-5.3) of hypoglycemic coma and 0.6 events/100 PY (0.4-0.8) of ketoacidosis.

### Dyslipidemia and Lipid-Lowering Treatment

**Figure 3:**
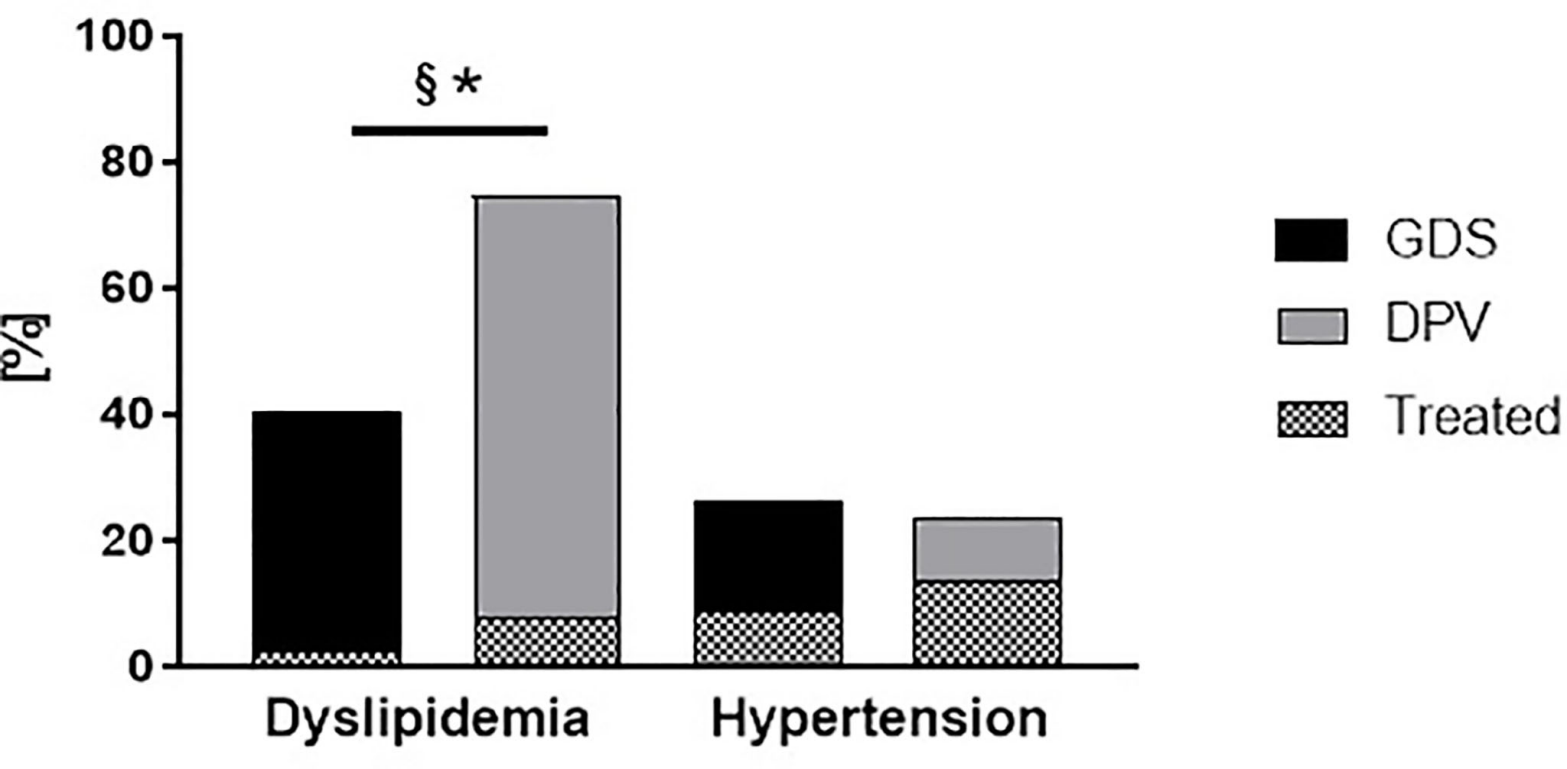
Prevalence and management of hypertension and dyslipidemia in patients with newly diagnosed type 1 diabetes from the GDS and DPV cohorts. Prevalence and management of modifiable cardiovascular risk factors (dyslipidemia and hypertension) in patients with newly diagnosed type 1 diabetes from the GDS (black) and DPV (grey) cohort. ‘Treated’ refers to the frequency of lipid-lowering medication or antihypertensive, respectively. Data are expressed as percent from the total number of measured participants in each cohort. P values refer to comparison of unadjusted data. *p < 0.05 regarding the prevalence of comorbidities; ^§^p < 0.05 regarding the percentage of treated patients.

**Figure 4:**
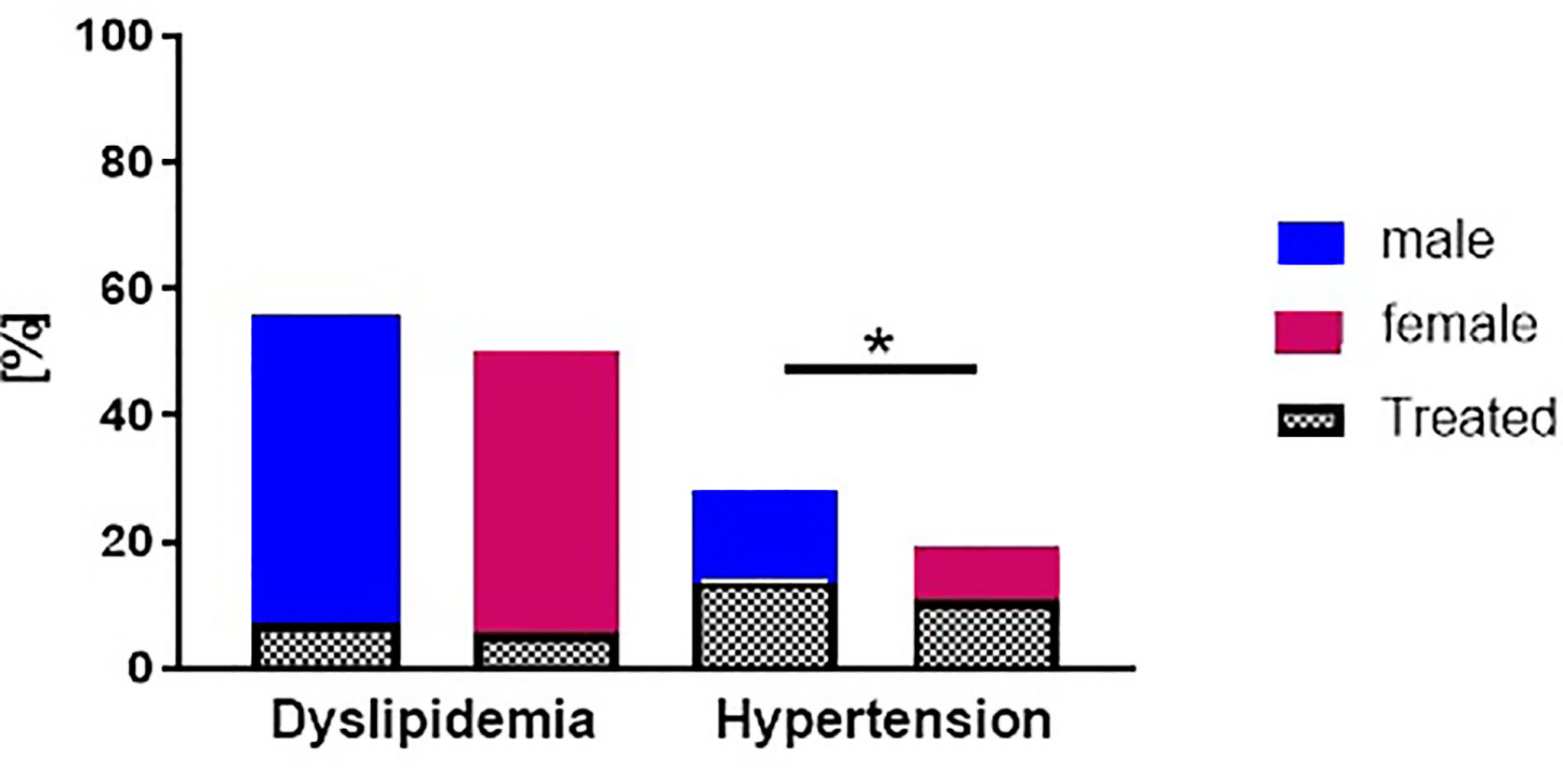
Prevalence and management of hypertension and dyslipidemia in patients with newly diagnosed type 1 diabetes stratified by sex. Prevalence and management of modifiable cardiovascular risk factors (dyslipidemia and hypertension) in males (blue) and females (red) with newly diagnosed type 1 diabetes from the GDS and DPV cohort. ‘Treated’ refers to the frequency of lipid-lowering medication or antihypertensive, respectively. Data are expressed as percent from the total number of measured participants in each cohort. P values refer to comparison of unadjusted data. *p < 0.05 regarding the prevalence of comorbidities.

Dyslipidemia, considering all cholesterol fractions and triglycerides, as defined above, afflicted 67% of the patients from both cohorts. There was a higher prevalence of dyslipidemia in the DPV cohort compared to the GDS cohort (77% vs 41%, p<0.001; **Figure 3**). When assessing the stratified ESC criteria for LDL cholesterol only (19), 40% of patients of the GDS and 47% of the DPV met the targeted levels. Of DPV patients, 7% had lipid-lowering medication compared to GDS where 2% had lipid-lowering medication (p<0.05). There was no difference in the prevalence of dyslipidemia between sexes (**Figure 4**).

### Hypertension and Antihypertensive Treatment

Hypertension was present in 398 (24%) of the patients with type 1 diabetes from both cohorts with no difference between both cohorts (p=0.39, **Figure 3**). Of the patients in the DPV cohort, merely 13% had antihypertensive medication compared to the GDS participants of whom 9% had antihypertensive medication.

**Figure 5:**
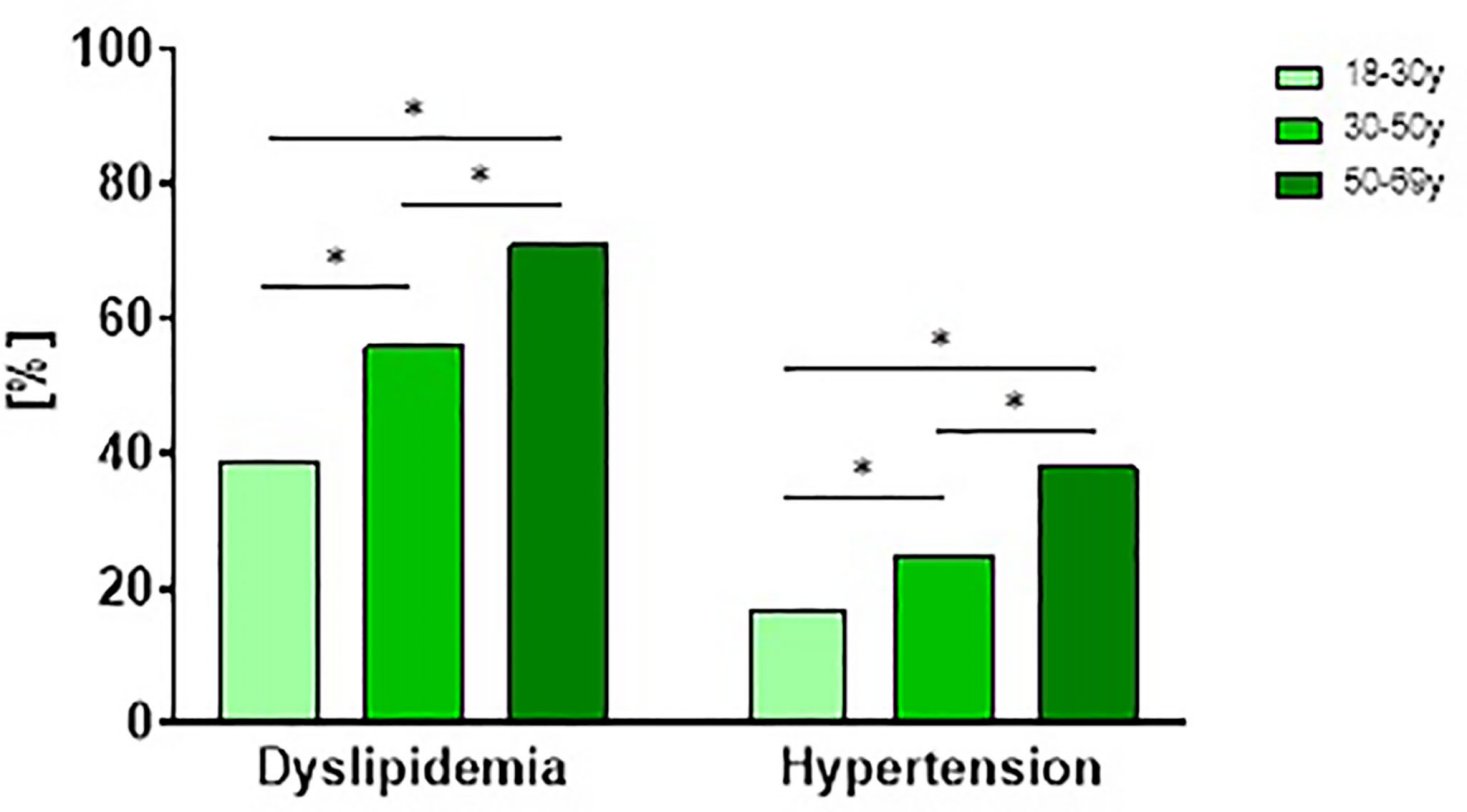
Prevalence and management of hypertension and dyslipidemia in patients with newly diagnosed type 1 diabetes stratified by age. Prevalence and management of modifiable cardiovascular risk factors (dyslipidemia and hypertension) stratified by age tertiles in participants with newly diagnosed type 1 diabetes from the GDS and DPV cohort. P values refer to comparison of unadjusted data. *p < 0.05 regarding the prevalence of comorbidities.

Men more frequently exhibited elevated blood pressure compared to women (p<0.001, **Figure 4**). In age-stratified analyses we observe an increase in prevalence of dyslipidemia and hypertension with age (**Figure 5**)

### Diabetes-Related Complications

**Figure 6:**
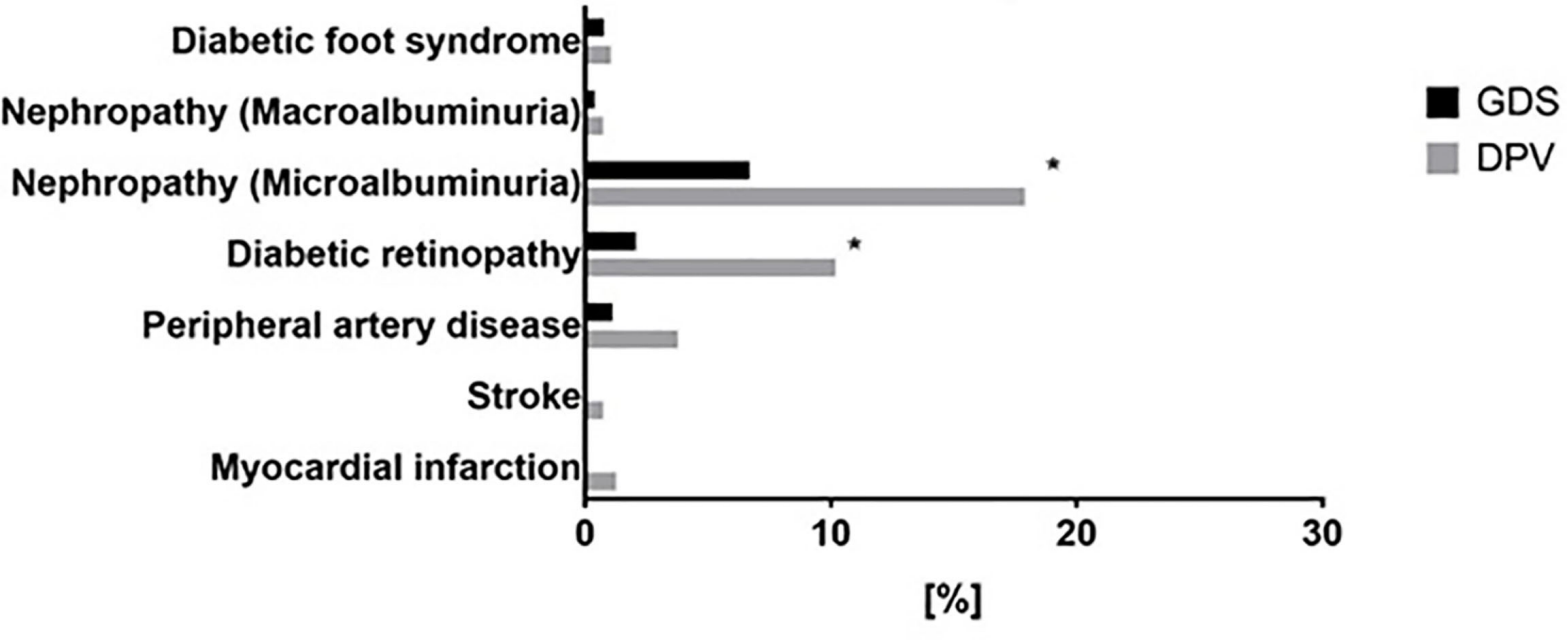
Prevalence of diabetes related complications in patients with newly diagnosed type 1 diabetes from the GDS and DPV cohorts. Prevalence of diabetes-related complications in patients with newly diagnosed type 1 diabetes from the GDS (black) and DPV (grey) cohort. Data was collected on the prevalence of diabetic foot syndrome, diabetic nephropathy (micro- or macroalbuminuria), diabetic retinopathy, peripheral artery disease, myocardial infarction and stroke. Data are expressed as percent from the total number of measured participants in each cohort. P values refer to data adjusted for age, sex, BMI, migration background, hypertension and dyslipidemia for microalbuminuria, diabetic retinopathy, peripheral artery disease and myocardial infarction. P values for macroalbuminuria and diabetic foot syndrome are unadjusted because of the low case numbers. *p < 0.05.

**Figure 6** shows the prevalence of diabetes-related complications adjusted for age, sex, BMI, and migration background. There was a higher prevalence of both micro- and macrovascular complications in the DPV compared to the GDS cohort. Of note, largest differences were observed for nephropathy (18% vs. 7%) and diabetic retinopathy (10% vs 3%) (**Figure 6**). Additional adjustment for dyslipidemia and hypertension did not affect these results.

Regression analyses revealed that hypertension was positively associated with nephropathy (β=0.52, p=0.01) but neither hypertension nor dyslipidemia associated with the prevalence of retinopathy (p=0.17 and p=0.79, respectively).

**Figure 7:**
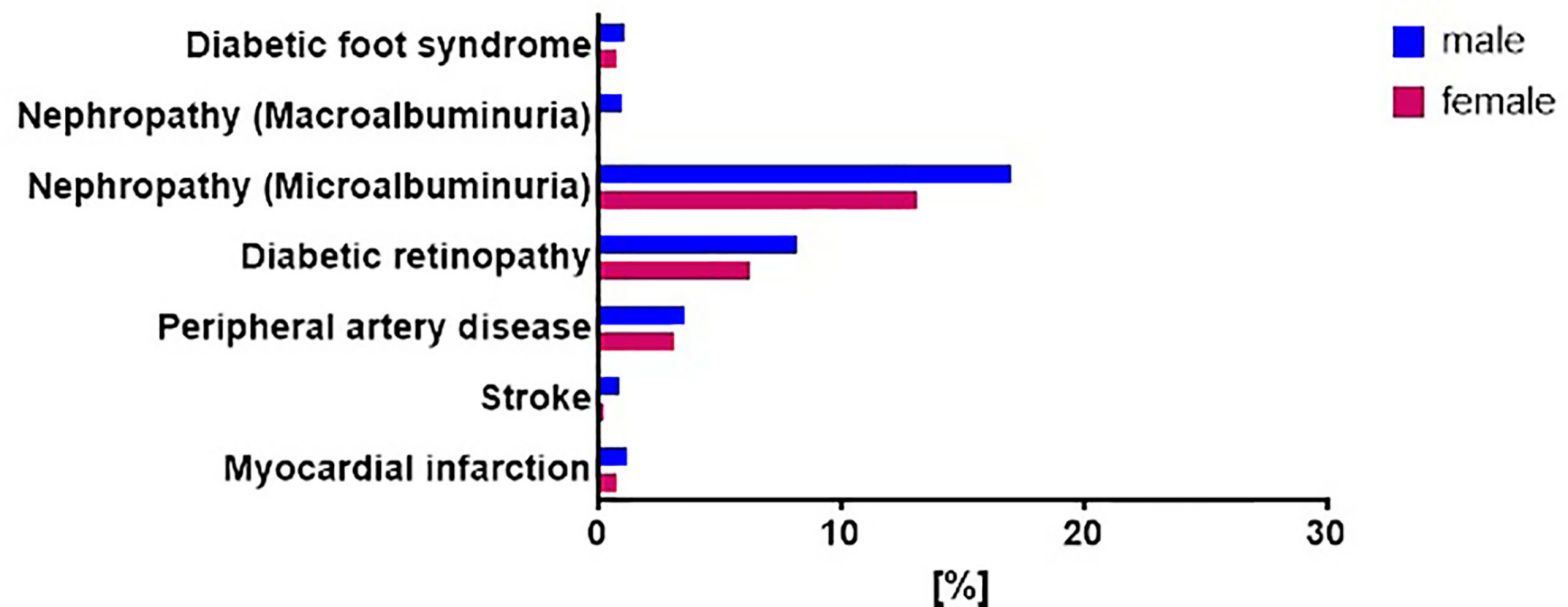
Prevalence of diabetes related complications in patients with newly diagnosed type 1 diabetes stratified by sex. Prevalence of diabetes-related complications in males (blue) and females (red) with newly diagnosed type 1 diabetes from the GDS and DPV cohort. Data was collected on the prevalence of diabetic foot syndrome, diabetic nephropathy (micro- or macroalbuminuria), diabetic retinopathy, peripheral artery disease, myocardial infarction and stroke. Data are expressed as percent from the total number of measured participants in each cohort. P values refer to data adjusted for age, sex, BMI, migration background, hypertension and dyslipidemia.*p < 0.05.

Sex-specific analyses for diabetes related complications revealed no differences in the prevalence of retinopathy, nephropathy or peripheral artery disease after adjustments for age, BMI, migration background, dyslipidemia and hypertension (**Figure 7**). The low number of cases of other complications did not allow sex-specific evaluation.

**Figure 8:**
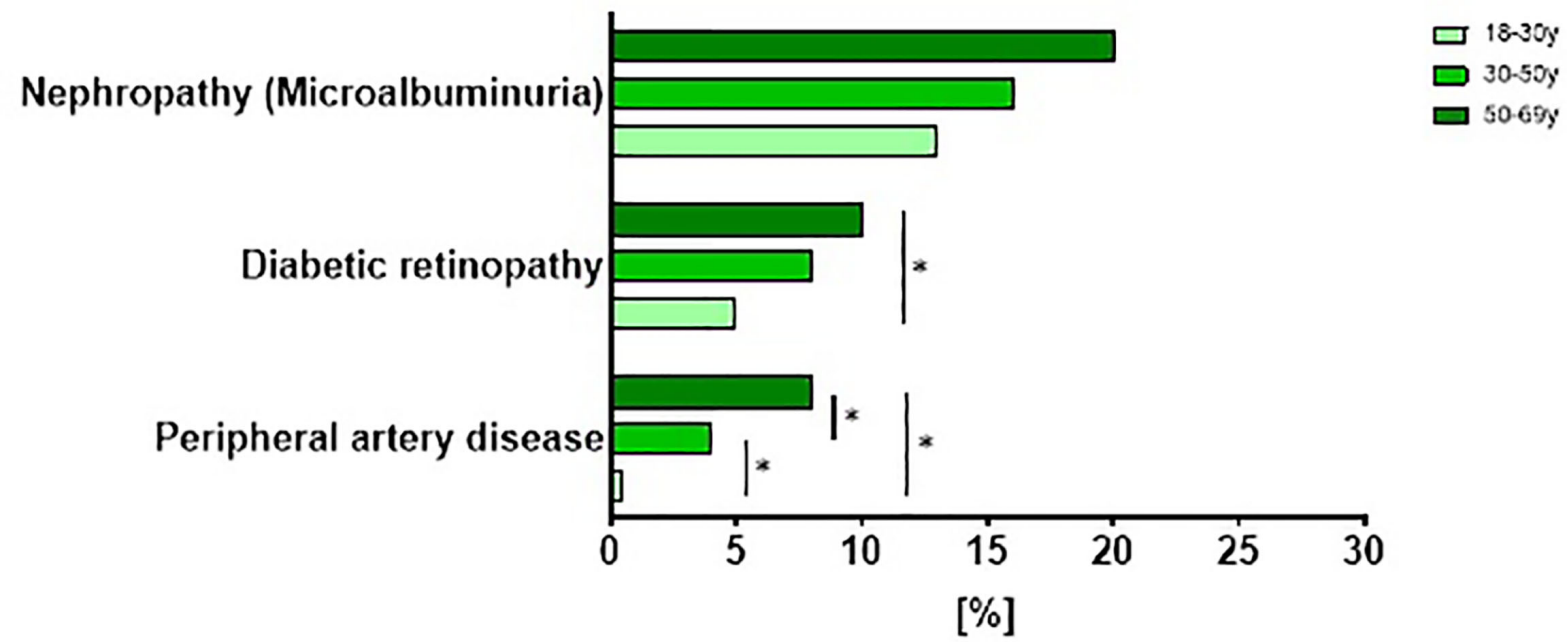
Prevalence of diabetes related complications in patients with newly diagnosed type 1 diabetes stratified by age. Prevalence of diabetes-related complications stratified by age tertiles in participants with newly diagnosed type 1 diabetes from the GDS and DPV cohort. Data are expressed as percent from the total number of measured participants in each cohort. *p < 0.05 regarding the prevalence of complications adjusted for age, sex, BMI, migration background, hypertension and dyslipidemia.

Similarly, additional analyses were performed to address age-specific differences in the prevalence of retinopathy, nephropathy or peripheral artery disease. After adjustments for age, sex, BMI, migration background, hypertension and dyslipidemia the differences between groups with regard to nephropathy did not reach statistical significance, however the prevalence of retinopathy and peripheral artery disease increased with age (**Figure 8**).

## Discussion

The present study assessed the prevalence of diabetes-related complications in patients with newly diagnosed type 1 diabetes from two different types of cohorts – voluntary participants of a prospective longitudinal study and patients of a population-based patient registry. Both cohorts show on average very good glucometabolic control, but inadequate management of risk factors such as hypertension and dyslipidemia.

This comparative analysis of data from two different types of cohorts provides effective information on the distribution of risk factors and complications and identifies common factors, independent of study design. Subsequently, this analysis may improve the generalizability of data deriving from clinical trials to the general diseased population.

Glucose homeostasis was assessed by fasting blood glucose and HbA1c levels as parameters to determine the effectiveness of diabetes management. Even though the same inclusion criteria were applied for both cohorts, patients of the GDS had a slightly lower HbA1c and fasting glucose compared to patients of the DPV. Therefore, our data aligns with previous studies which suggested that patients voluntarily participating in clinical studies might be more inclined towards preserving a good glycemic control. This may be related to the opportunity to ask investigators for advice and recommendations, regular monitoring, as well as increased awareness and access to information compared to standard care (22). Other differences may reside in educational and social background. Some individuals with type 1 diabetes had no insulin treatment after diagnosis, possibly accounting for cases of preserved beta cell function in autoimmune diabetes (23) or may be related to partial remission (24) within the short time frame after diagnosis. Moreover, 14% of type 1 diabetes patients also received oral glucose lowering diabetes-medication. Metformin had been shown to reduce the insulin dose requirement in insulin resistant individuals, insulin-induced weight gain, and cholesterol levels (25). Therefore, recent studies focused on the assessment of the effects of metformin on the cardiovascular system in adults with type 1 diabetes. The results of these investigations suggest a possible role of metformin in the prevention of cardiovascular events in type 1 diabetes by reducing body weight and LDL-cholesterol levels (26). The addition of the glucagon-like peptide 1 (GLP-1) receptor agonists or of a sodium–glucose cotransporter 2 (SGLT2) inhibitor to insulin therapy was shown to trigger small reductions in HbA1c compared with insulin alone in people with type 1 diabetes and also reduced body weight (27–29). However, SGLT2 inhibitor use is also associated with adverse events including ketoacidosis (30), and was only prescribed off-label for patients with type 1 diabetes, as was also the case for GLP-1 receptor agonists, at the time of data acquisition in Germany, which could explain the low prescription frequency for newly diagnosed type 1 diabetes.

We further underline the need for multidisciplinary interventions focusing on groups at high risk for hyperglycemic crises in order to prevent ketoacidotic events. Diabetic ketoacidosis remains a serious endocrine emergency, associated with morbidity and mortality. Younger age, ethnicity, low income, and poor glycemic control were associated with an increased risk of hyperglycemic crises (31). Recent studies further these findings by analyzing the rate of readmission for diabetic ketoacidosis in type 1 diabetes. Multiple concomitant complications, hypertension, female sex, and incompliance towards medical advice were significant predictors of readmission (32).

In both cohorts, dyslipidemia was highly prevalent in type 1 diabetes and management of lipid abnormalities was suboptimal according to guidelines (33). The apparent inadequate treatment of these patients may have serious implications for the subsequent development of diabetes-related complications. The American Diabetes Association recommends lipid-lowering pharmacotherapy for patients with concomitant cardiovascular risk factors such as elevated LDL cholesterol, high blood pressure, smoking, obesity, and/or family history of premature cardiovascular events (33). In spite of a prevalence of dyslipidemia in 77% of the DPV patients and 41% of the GDS participants, only 7% and 2% were treated with lipid-lowering agents, respectively. Along these lines, only 40% of patients with diabetes of GDS and 47% of DPV met the stratified LDL cholesterol targets as defined by the ESC (19).

Hypertension, together with reduced insulin sensitivity and dyslipidemia, can contribute to an increased cardiovascular risk (34) in patients with diabetes. Nevertheless, only 8% of patients diagnosed with hypertension in the DPV cohort and 14% of those in the GDS cohort had antihypertensive medication, pointing to a considerable therapeutic inertia in the management of modifiable risk factors. Our study is in accordance to a previous comparison of the DPV registry with the American T1D Exchange Clinic Network showing that individuals with type 1 diabetes are inadequately treated for hypertension and dyslipidemia (35). Clinical inertia was discussed as one potential reason for the observed undertreatment while the authors emphasized the importance to strengthen physician and patient education on early treatment of cardiovascular risk factors. However, while we included individuals with recent-onset type 1 diabetes, Shah et al. only included individuals with type 1 diabetes aged ≥12 years and a diabetes duration of at least one year.

Socioeconomic factors and sex may have an impact on the adherence to lipid-lowering therapy as previous studies suggest that in individuals with type 1 diabetes lower adherence was associated with male sex, younger age, marital status and country of birth (36).

A previous study has also shown an increased prevalence of diabetes-related complications in the DPV cohort compared to an American registry (37), which was explained by a more frequent use of antihypertensive drugs and/or lower blood pressure of the 3297 participants aged > 60 years. This is also seen for the present comparison at a national level between the DPV and the GDS cohort with regard to both micro- and macrovascular complications. Nevertheless, it should be noted that the prevalence of dyslipidemia in individuals with type 1 diabetes varies markedly by country and age of the participants, with reported values between 3.8–72.5% (38). Initiating good glycemic control and strict monitoring of modifiable risk factors from onset of diabetes, irrespective of diabetes type, is essential to counteract pathogenetic processes causative for micro- and macrovascular complications (39). In this study, retinopathy and nephropathy, were more frequent compared to classical macrovascular complications such as myocardial infarction or stroke, irrespective of study cohort.

Diabetic retinopathy represents a frequent microvascular complication of diabetes and a leading cause of blindness among adults (40). In addition to hyperglycemia, dyslipidemia and hypertension may contribute to the onset and progression of diabetic retinopathy (41). Previous studies in type 1 diabetes showed that intensified glycemic control was highly effective in reducing the risk of developing microvascular complications (42). In the present analysis, in spite of predominantly good metabolic control and short diabetes duration, up to 10% of type 1 diabetes patients presented with diabetic retinopathy within the first year after diagnosis. As with peripheral artery disease, the prevalence of retinopathy increased with age, in line with previous observations showing that not only duration of diabetes but also advancing age independently predicts diabetes morbidity rates (43).

Nephropathy, another major microvascular complication, was linked to impaired glycemic control, increased body weight, and improper insulin dosage (44). Our study showed that microalbuminuria was present in 7% of the GDS population and was twice more frequent in DPV. As expected, microalbuminuria was positively associated with hypertension. Nevertheless, generally, it appears that renal dysfunction and hyperglycemia have improved considerably during recent years for people with type 1 diabetes. This finding has important implications for quality of life, health economics and prognosis regarding cardiovascular mortality (45).

Cardiovascular disorders are a major macrovascular complication which can increase the morbidity and mortality in patients with diabetes (46). However, evidence of cardiovascular risks and management of type 1 diabetes is often extrapolated from studies in type 2 diabetes patients (47). Overall, cardiovascular events are more common and occur earlier in patients with type 1 diabetes patients than in nondiabetic populations, with an even higher discrepancy in the female population (33). The prevalence of cardiovascular disease in patients with type 1 diabetes varies substantially based on disease duration, age of cohort, and sex, as well as possibly by ethnicity (9).

Factors related to sex seem to explain the consistently observed increased risk of incident type 1 diabetes for males of all age categories (48). However, females with type 1 diabetes are more likely to develop complications (9), possibly due to risk factors such as centrally distributed adipose tissue, which may contribute to their relatively higher risk for cardiovascular disease. The results of the present study did not reveal any differences in microvascular complication between male and female patients with type 1 diabetes. However, likely due to the short disease duration, patients with type 1 diabetes rarely presented with overt cardiovascular disease. Further mechanistic insights with regard to possible metabolic risk factors were limited due to the low number of cases.

The present comparative study may suffer from a certain degree of selection bias, since participants of the GDS are generally compliant, health-conscious patients with diabetes with an HbA1c below 9%. This could trigger a ‘healthy user bias’, whereas the DPV sample could be prone to the Berkson’s fallacy, since a considerable fraction of the study population is selected from hospital care and therefore may be less healthy than the general population (49). This provides a sample population that may not be representative of the general population of persons with type 1 diabetes.

While for the GDS the baseline characteristics and blood chemistry have been done using standardized operating procedures in a centralized laboratory facility, this was not the case for patients of the DPV. Therefore, variations in sample collection and handling are to be expected between centers, and it cannot be guaranteed that DPV samples were collected in fasted conditions. Furthermore, for the GDS cohort the presented parameters are routinely screened in all study participants, while in the DPV some parameters are only measured if there is clinical suspicion (e.g. thyroid function, diabetes-related antibodies), increasing the likelihood of pathological levels. Recall bias cannot be excluded when addressing self-reported data. Also, other mediating factors that are not routinely quantified but may impact the results, such as physical activity behavior or smoking, could not be comprehensively assessed in the context of the present analysis.

### Conclusion

The present analysis of data from different German diabetes cohorts allows for a better understanding of the distribution of risk factors and their association to diabetes-related complications and renders similar results irrespective of study design. The current mismanagement of known modifiable risk factors possibly contributes to the development of diabetes-related complications. We observed that diabetic nephropathy and retinopathy are the most frequent complications in type 1 diabetes affecting up to 10% of the patients within the first year after diagnosis. There were no differences in the prevalence of microvascular complication between male and female patients in our study. These observations warrant intensified and targeted treatment of modifiable risk factors from disease onset in order to prevent development and progression of diabetes-related complications.

## Data Availability Statement

The raw data supporting the conclusions of this article will be made available by the authors, without undue reservation.

## Ethics Statement

The studies involving human participants were reviewed and approved by ethics boards of the Medical Faculty of the Heinrich Heine University Düsseldorf (reference number 4508). The patients/participants provided their written informed consent to participate in this study.

## Author Contributions

OZ wrote the manuscript and researched data. SL, JR, RH performed the statistical analyses. WK, SM, MH, DR, SL, JR, JS, KM, VB, MR, RH researched data, contributed to the discussion and reviewed/edited the manuscript. All authors critically reviewed the manuscript. JS and RH are the guarantors of this work and, as such, had full access to all the data in the study and take responsibility for the integrity of the data and the accuracy of the data analysis. All authors read and approved the final manuscript.

## Funding

The German Diabetes Study was initiated and financed by the German Diabetes Center (DDZ), which is funded by the German Federal Ministry of Health (BMG) and the Ministry of Culture and Science of the state North Rhine-Westphalia and from the German Federal Ministry of Education and Research (BMBF) to the German Center for Diabetes Research (DZD) and the Schmutzler Stiftung, Germany. DPV is supported through the German Center for Diabetes Research (FKZ: 82DZD14A02). A listing of sites contributing data for the DPV is available at http://www.d-p-v.eu.

## Conflict of Interest

The authors declare that the research was conducted in the absence of any commercial or financial relationships that could be construed as a potential conflict of interest.

## Publisher’s Note

All claims expressed in this article are solely those of the authors and do not necessarily represent those of their affiliated organizations, or those of the publisher, the editors and the reviewers. Any product that may be evaluated in this article, or claim that may be made by its manufacturer, is not guaranteed or endorsed by the publisher.
